# Modulation of amygdala reactivity following rapidly acting interventions for major depression

**DOI:** 10.1002/hbm.24895

**Published:** 2020-03-01

**Authors:** Joana R. A. Loureiro, Amber Leaver, Megha Vasavada, Ashish K. Sahib, Antoni Kubicki, Shantanu Joshi, Roger P. Woods, Benjamin Wade, Eliza Congdon, Randall Espinoza, Katherine L. Narr

**Affiliations:** ^1^ Department of Neurology Ahamason‐Lovelace Brain Mapping Center Los Angeles California; ^2^ Northwestern University Clinical and Translational Sciences Institute (NUCATS) Chicago Illinois; ^3^ Department of Psychiatry and Biobehavioral Sciences University of California Los Angeles Los Angeles California

**Keywords:** amygdala, ECT, emotion processing, ketamine, MDD, tfMRI

## Abstract

Electroconvulsive therapy (ECT) and ketamine treatment both induce rapidly acting antidepressant effects in patients with major depressive disorder unresponsive to standard treatments, yet their specific impact on emotion processing is unknown. Here, we examined the neural underpinnings of emotion processing within and across patients (*N* = 44) receiving either ECT (*N* = 17, mean age: 36.8, 11.0 *SD*) or repeated subanesthetic (0.5 mg/kg) intravenous ketamine therapy (*N* = 27, mean age: 37.3, 10.8 *SD*) using a naturalistic study design. MRI and clinical data were collected before (TP1) and after treatment (TP2); healthy controls (*N* = 31, mean age: 34.5, 13.5 *SD*) completed one MRI session (TP1). An fMRI face‐matching task probed negative‐ and positive‐valence systems. Whole‐brain analysis, comparing neurofunctional changes within and across treatment groups, targeted brain regions involved in emotional facial processing, and included regions‐of‐interest analysis of amygdala responsivity. Main findings revealed a decrease in amygdalar reactivity after both ECT and ketamine for positive and negative emotional face processing (*p* < .05 family wise‐error (FWE) corrected). Subthreshold changes were observed between treatments within the dorsolateral prefrontal cortex and insula (*p* < .005, uncorrected). BOLD change for positive faces in the inferior parietal cortex significantly correlated with overall symptom improvement, and BOLD change in frontal regions correlated with anxiety for negative faces, and anhedonia for positive faces (*p* < .05 FWE corrected). Both serial ketamine and ECT treatment modulate amygdala response, while more subtle treatment‐specific changes occur in the larger functional network. Findings point to both common and differential mechanistic upstream systems‐level effects relating to fast‐acting antidepressant response, and symptoms of anxiety and anhedonia, for the processing of emotionally valenced stimuli.

## 1 INTRODUCTION

Major depressive disorder (MDD) is the largest contributor to disability worldwide, affecting >300 million people (WHO, 2019), and leads to many lives lost to suicide (Mrazek, Hornberger, Altar, & Degtiar, 2014). Standard pharmacotherapies for depression mostly target monoaminergic neurotransmission. However, first‐line antidepressants work relatively slowly (over several weeks to months), and ~60% of patients fail to achieve remission with initial medication, while approximately one‐third fail to respond to 2 or more medication trials (Gaynes et al., 2009; Trivedi et al., 2006). In such patients, who are described as having treatment resistant depression (TRD; Nemeroff, 2007), electroconvulsive therapy (ECT) has higher response and remission rates and a more rapid onset of action than standard antidepressants. However, ECT carries stigma and fear of cognitive side effects (Verwijk et al., 2012).

Ketamine is a noncompetitive *N*‐methyl‐d‐aspartate (NMDA) receptor antagonist, which when administered at low concentration, is also found to produce fast‐acting (within hours) antidepressant effects (Berman et al., 2000; Zarate Jr. et al., 2006). Though the clinical effects of a single ketamine treatment typically diminish over subsequent days, two to three times weekly administration results in a more sustained response lasting up to a month and longer (Murrough et al., 2013; Shiroma et al., 2014; Singh et al., 2016). The pronounced antidepressant effects of ketamine suggest an alternative to the monoamine deficiency hypothesis of depression, prompting new research to understand its therapeutic mechanisms (Duman, Aghajanian, Sanacora, & Krystal, 2016).

Though the molecular mechanisms triggering rapid response to ECT and ketamine interventions likely differ due to distinct electrical or pharmacological perturbation of the central nervous system, antidepressant effects are expected to converge at the brain systems level to account for changes in mood and behavior. The processing of emotional human faces is a key aspect of social function, and processing biases (e.g., interpreting neutral faces as sad, or happy faces as neutral; Diener et al., 2012; Groenewold, Opmeer, de Jonge, Aleman, & Costafreda, 2013) are strong determinants of interpersonal aspects of depressive illness (Bourke, Douglas, & Porter, 2010; Stuhrmann, Suslow, & Dannlowski, 2011). Understanding how ECT and ketamine modulate emotion processing may thus provide new insights into the neural correlates of rapid clinical response.

Interacting neural circuits including the amygdala, orbitofrontal cortex, and striatum are centrally involved in emotional identification and production, while the dorsolateral prefrontal (DLPFC) and anterior cingulate cortex (ACC) and connected regions appear important for emotion regulation (Phillips, Drevets, Rauch, & Lane, 2003; Rive et al., 2013). Prior depression fMRI studies of emotional processing including negative‐valenced faces typically report hyper‐responsivity within the amygdala and striatal regions, and fusiform, cingulate, and insula cortex in patients relative to controls. Concurrently, reduced activation is observed in prefrontal emotional regulation regions (Groenewold et al., 2013). Though meta‐analysis finds only increased cingulate cortex activity (Groenewold et al., 2013) for positive emotional faces, hypoactivation in limbic/striatal regions are observed in independent studies (Epstein et al., 2006; Keedwell, Andrew, Williams, Brammer, & Phillips, 2005).

Meta‐analyses addressing the effects of repeated antidepressant drug therapy on neural activity during the processing of emotionally valenced stimuli support that functional neuroplasticity occurs in limbic (amygdala, hippocampus), prefrontal (ACC, DLPFC), insular and occipital/temporal regions in relation to treatment (Delaveau et al., 2011; Ma, 2015). However, the type of pharmacotherapy and functional probe (positive/negative, facial/nonfacial), influences the pattern and direction of effects. Only three published studies, including one in the same cohort, have investigated how ketamine targets brain circuits involved in emotion processing (Murrough et al., 2015; Reed et al., 2018, 2019). Specifically, a placebo‐controlled double‐blind trail of single ketamine versus saline using an implicit and explicit facial recognition functional task showed reductions in neural activity post‐ketamine in patients relative to controls in frontal, temporal, and posterior cingulate regions (Reed et al., 2019). In the same sample, normalization of medial prefrontal activity post‐ketamine was also observed during an attentional bias dot probe task with emotional face stimuli (Reed et al., 2018). Using a facial emotion recognition task, a separate study reported a normalization of reduced activity for positive faces in the caudate following single ketamine (Murrough et al., 2015). No study has yet examined the effects of repeated ketamine therapy. For ECT, a study investigating amygdala reactivity during subliminally presented negatively valenced face stimuli, showed normalization of amygdala hyper‐reactivity for sad faces after both ECT and medication treatment.

Although the existing literature suggests that different antidepressants modulate aspects of functional circuitry involved in emotional processing, no study to date has investigated whether repeated ketamine treatment and ECT, which both elicit pronounced and rapidly acting clinical effects, perturb overlapping, or distinct neural circuitry. Using optimized image acquisition methods (Barch et al., 2013; Glasser et al., 2013) and a well‐validated emotional face‐matching task targeting amygdala function and corticolimbic circuitry (Chai et al., 2015), we thus evaluated the neural effects of serial ketamine therapy and ECT for emotional processing. Here, TRD patients received MRI and clinical assessments before and after receiving an index series of ECT, or after four ketamine treatments. Analyses addressed main effects of treatment irrespective of modality, and within and between treatment effects within the facial emotion processing network, and amygdala regions‐of‐interest. Follow‐up analyses addressed if changes in neural response associate with changes in mood, anxiety, and anhedonia. Cross‐sectional differences between patients and controls at baseline were also examined.

Based on prior findings for ECT and medication treatment (Redlich et al., 2017), we predicted reductions in BOLD activity in the amygdala would occur for both treatments for negative stimuli. Though encompassing different brain activation paradigms, based on meta‐analysis of standard pharmacological treatments (Delaveau et al., 2011; Ma, 2015), single ketamine administration in depression (Murrough et al., 2015; Reed et al., 2019) and in healthy subjects (Scheidegger et al., 2016; Scheidegger et al., 2016), we hypothesized that treatment‐related changes in neural activity would also occur in the larger corticolimbic‐striatal face emotion processing network, including in striatal, medial prefrontal/ACC regions, and insula for negative stimuli, and increases in medial prefrontal/ACC regions for positive stimuli. Since ketamine and ECT have different molecular mechanisms of action, are subject to different side effects and may target different symptoms, we also expected that some neural effects would diverge at the systems level especially in regions involved in cognition and memory (Li et al., 2018; Perrin et al., 2012; Yrondi, Peran, Sauvaget, Schmitt, & Arbus, 2018).

## 2 METHODS AND MATERIALS

### 2.1 Participants and study design

Participants included 32 nondepressed healthy controls (HCs) and 44 individuals experiencing a major depressive episode evaluated by the Structured Clinical Interview for DSM‐V (First, Williams, Karg, Spitzer, & American Psychiatric Association Publishing, 2016). Patients were defined as TRD (i.e., failed ≥2 adequate antidepressant trials in the current episode and had been continuously depressed for ≥6 months) based on clinician interview and the antidepressant treatment history form (Sackeim, 2001). To evaluate emotional processing within and across fast‐acting therapies, 27 TRD patients received serial ketamine infusion and 17 patients received an index series of ECT using a nonrandomized naturalistic design. All patients had moderate to severe depressive symptoms as determined the Hamilton depression rating scale (HDRS), 17‐item (Hamilton, 1960; scores ≥18), and did not differ in terms of disease severity and history (Table 1). See Supporting Information for detailed inclusion/exclusion criteria.

**Table 1:** Participants demographic information with statistical differences at baseline, and clinical measures for both patient groups (ECT and ketamine) for baseline (TP1) and for after treatment (TP2; 24 to 72 hr after the fourth infusion of ketamine and after the last ECT session)

		ECT			Ketamine		HC		
		(*N* = 17)			(*N* = 27)		(*N* = 32)	MDD VS HC	ECT VS Ket.
	TP1	TP2		TP1	TP2	TP1 VS TP2	TP1	(TP1)	(TP1)
Feature	Mean (SD)	Mean (SD)	TP1 VS TP2	Mean (SD)	Mean (SD)		Mean (SD)		
Age	36.82 (11.02)	N/A	N/A	37.26 (10.82)	N/A	N/A	34.45 (13.51)	*T* = 1.09, *p* = .28	*T* = 0.32, *p* = .75
Sex (% female)	71	N/A	N/A	41	N/A	N/A	59	*X* = 0.26, *p* = .61	*X* = 3.31, *p* = .07
Education (years)	10.02 (1.89)	N/A	N/A	9.37 (3.43)	N/A	N/A	10 (2.10)	*T* = −1.28, *p* = .21	*T* = −4.58, *p* = .65
Duration lifetime illness (years)	19.29 (10.61)	N/A	N/A	19.31 (12.73)	N/A	N/A	N/A	N/A	*T* = 0.17, *p* = .86
Nr. depressive episodes	4 (3.81)	N/A	N/A	3.38 (2.94)	N/A	N/A	N/A	N/A	*T* = 0.39, *p* = .70
Age of onset (years)	18.06 (9.18)	N/A	N/A	18 (9.56)	N/A	N/A	N/A	N/A	*T* = −0.02, *p* = .98
Current episode (years)	2.62 (4.14)	N/A	N/A	6.50 (7.69)	N/A	N/A	N/A	N/A	*T* = 2.02, *p* = .05
Generalized anxiety	11	N/A	N/A	18	N/A	N/A	N/A	N/A	N/A
Mood disorders	7	N/A	N/A	0	N/A	N/A	N/A	N/A	N/A
Manic episodes	4	N/A	N/A	0	N/A	N/A	N/A	N/A	N/A
Feeding and eating disorder	4	N/A	N/A	1	N/A	N/A	N/A	N/A	N/A
Substance use disorder	12	N/A	N/A	8	N/A	N/A	N/A	N/A	N/A
Trauma and stressor related disorders	9	N/A	N/A	6	N/A	N/A	N/A	N/A	N/A
ADHD	0	N/A	N/A	1	N/A	N/A	N/A	N/A	N/A
Somatic symptom and related disorders	0	N/A	N/A	1	N/A	N/A	N/A	N/A	N/A
HDRS	21.41 (8.33)	15.35 (8.60)	*T* = 3.07, *p* < .01	20.15 (4.70)	8.93 (4.46)	*T* = 10.73, *p* < 0.01	N/A	N/A	*T* = −0.57, *p* = .57
DASS	7.82 (5.60)	6.53 (4.38)	*T* = 1.28, *p* = .22	5.52 (5.32)	1.67 (2.20)	*T* = 5.51, *p* < .01	N/A	N/A	*T* = −1.37, *p* = .18
SHAPS	6.76 (4.41)	3.12 (3.89)	*T* = 6.08, *p* < .01	8.07 (3.88)	3.81(4.15)	*T* = 5.07, *p* < .01	N/A	N/A	*T* = 1.03, *p* = .31

*Note*: Comorbidities are reported as counts.

Abbreviations: DASS, depression anxiety stress scale; ECT, electroconvulsive therapy; HC, healthy controls; HDRS, Hamilton depression rating scale; MDD, major depressive disorder; SHAPS, Snaith‐Hamilton pleasure scale; TP1, baseline time‐point; TP2, posttreatment time‐point; VS, voxel size.

**Figure 1:**
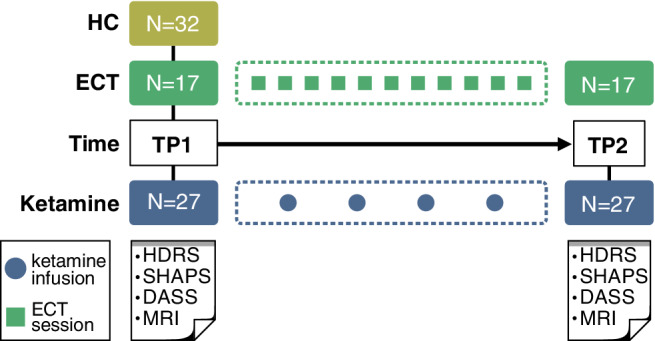
Study design showing the MRI sessions and clinical scales acquired at each time point

Patients received MRI scanning and clinical assessments at two time points: (a) pretreatment baseline (TP1) occurring within 1‐week of the first ECT or ketamine treatment; and (b) 24–72 hr after the last ketamine infusion or within a week of completing the ECT index series (TP2; Figure 1). HCs were assessed at a single time‐point (TP1). All subjects provided written informed consent following procedures approved by the UCLA Institutional Review Board (IRB).

At each time point, depression severity was assessed using the HDRS (Hamilton, 1960; Koo, Han, Park, & Kwon, 2017). Since anxiety affects amygdala responsivity (Beesdo et al., 2009; Stein, Simmons, Feinstein, & Paulus, 2007), the Depression Anxiety Stress Scale (DASS) (Lovibond, 1995) was also administered. Anhedonia, linked with altered reward‐related processing (Russo & Nestler, 2013), was measured with the Snaith–Hamilton pleasure scale (SHAPS; Snaith et al., 1995). Psychiatric comorbidities for ketamine and ECT patients were assessed (Table 1), and measures of ketamine side effects were also acquired using the Clinician‐Administered Dissociative States Scale (CADSS) after 60 min of each infusion (mean = 1.12, *SD* = 2.42).

### 2.2 Ketamine treatment

Patients received ketamine infusions 2–3 days apart (2–3× a week) for a total of four infusions. At each session, performed as an outpatient procedure, a single subanesthetic dose (0.5 mg/kg) of ketamine diluted in 60 cc normal saline was delivered intravenously via pump over a 40‐minute period in a private room at the UCLA Clinical Research Center or Resnick Neuropsychiatric Hospital. Vital sign monitoring included blood pressure, pulse oximetry, and respiratory rate recording every 3 min and a continuous cardiac rhythm strip. Mental status monitoring assessed for any untoward behavioral or psychological effects. Ketamine patients were permitted to remain on stable (if unchanged for at least the preceding 6‐weeks), approved monoaminergic antidepressant therapy (i.e., selective serotonin and/or norepinephrine reuptake inhibitors [SSRIs and SNRIs], norepinephrine and dopamine reuptake inhibitors, serotonin antagonist and reuptake inhibitor [SARIs] and tricyclics) for the duration of the study (see Table S1). Benzodiazepines were discontinued at least 24 hours prior to all study visits (i.e., scans and treatment sessions).

### 2.3 ECT treatment

For ECT (5000Q MECTA Corp), seizure threshold was individually titrated at the first session. All patients received right‐unilateral ECT (pulse width: 0.3 ms, amplitude: 800 mA.) However, based on clinically determined rates of response, 48% of patients were subsequently switched to bitemporal ECT (pulse width: 0.5 ms, amplitude: 8,000 mA). ECT was also administered 2–3 days apart, and continued until patients achieved maximal response or remission for at least a week as evaluated by mood scales and assessment by expert ECT Psychiatrist. The length of the ECT index was individually prescribed (average number of sessions = 14).

### 2.4 Image acquisition and preprocessing

Imaging was performed on a Siemens 3T Prisma MRI system at UCLA's Brain Mapping Center using a 32‐channel head coil. Imaging sequences were identical to those used by the Human Connectome Project Lifespan studies for Aging and Development (https://www.humanconnectome.org). Structural scans included a T1‐weighed (T1w) multi‐echo MPRAGE and a T2‐weighted (T2w) acquisition (see Supporting Information for parameters). For functional scans, two runs of a multiband EPI sequence with opposite phase encoding directions were acquired (voxel size [VS] = 2 mm isotropic; repetition time [TR] = 800 ms; echo time [TE] = 37 ms, flip‐angle [FA] = 52°, MB accl. factor = 8; phase enc. direction = AP[run1]/PA[run2]; total acquisition time [TA] = 4:41 min [per run]).

Imaging data were preprocessed using the HCP minimal pipelines (Glasser et al., 2013) implemented within the BIDS‐App (Gorgolewski et al., 2017). After preprocessing, the functional images were further denoised using FSL's FIX (https://fsl.fmrib.ox.ac.uk/fsl/fslwiki/FIX). Smoothing (5 mm) was applied to the preprocessed images using the grayordinates‐based approach (Barch et al., 2013). Image quality was assessed with plots of relative and absolute motion and inspection of the ICA components for each subject. Data with = >3 mm of motion in any dimension and/or with artifacts after FIX processing were removed. Two subjects, not counted in the *N* = 76 sample size, were excluded (Marcus et al., 2013).

### 2.5 Emotional faces functional imaging task

**Figure 2:**
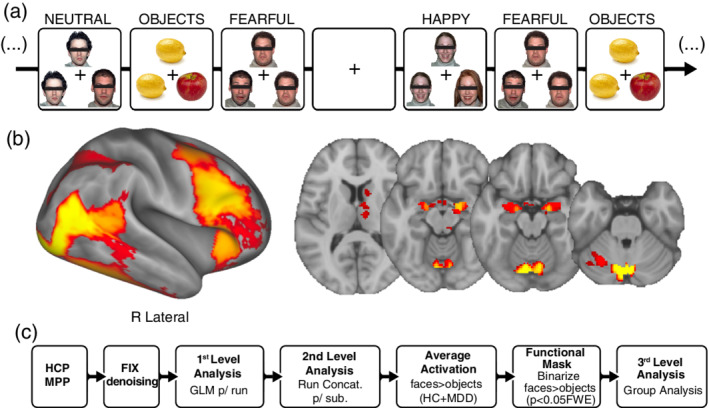
FMRI task, mean activation, and processing pipeline. (a) face‐matching task example sequence showing the four stimulus conditions; (b) Mean activation map obtained from the one sample *t*‐test of both MDD patients and HC at baseline; (c) Flow diagram of preprocessing and first and second level postprocessing steps. HC, healthy control; MDD, major depressive disorder

The functional task consisted of a validated affect‐labeling face‐matching blocked paradigm (Chai et al., 2015), adapted from (Hariri et al., 2002) and the HCP Lifespan face‐matching task (Barch et al., 2013) to include faces that exhibit fearful, happy, or neutral emotions, and objects (fruits or vegetables). During this task, participants selected which of two images displayed at the bottom of the screen matched a target image displayed at the top of the screen using a button box (Figure 2a).

### 2.6 Task‐fMRI analysis

For first and second‐level analysis of BOLD fMRI data, whole‐brain analysis used CIFTI file format (Glasser et al., 2013). First‐level fixed‐effects analysis fit a general linear model to each run separately with regressors for each of the four trial types (happy, fearful, and neutral faces and objects). Second‐level analysis included one sample *t*‐tests to estimate activation across runs for each first‐level contrast in each subject. To evaluate changes in the emotional face processing network while controlling for task demands (e.g., visual processing and attention), we explicitly focused on statistical contrasts comparing faces and objects (fearful > objects and happy > objects). Third‐level (i.e., higher‐level) analyses addressed treatment and group effects for the second‐level contrasts within brain regions involved in emotion regulation and face processing using a functional mask defined by the group activation map for the all‐faces > objects contrast (i.e., neutral + happy + fearful faces > objects) across all subjects thresholded at *p* < .05, FWE corrected (Figure 2b).

Higher‐level analyses tested for: (a) *Main effects of treatment* common to both treatment types (ECT and ketamine) by comparing pretreatment (TP1) and post‐treatment (TP2) using a paired *t*‐test, and performing follow‐up amygdala ROI analysis within each group, (b) *Differences in treatment‐related change across treatments* by comparing TP2–TP1 subtraction maps of second‐level contrasts (e.g., TP2 happy > objects minus TP1 happy > objects) with two‐sample *t*‐tests; (c) *Correlations between treatment‐related change and clinical response* again using TP2–TP1 subtraction maps of second‐level contrasts and percent change in HDRS, DASS, and SHAPS scores over treatment; and (4) *Cross‐sectional group effects* comparing HC and MDD patients at baseline (TP1) using a two‐sample *t*‐test. Age and sex were used as regressors of no interest for between‐subjects tests (#2 and #4 above).

FMRI task performance was evaluated for use as a potential covariate for longitudinal and cross‐sectional comparisons. Since significant main effects or interactions were absent (see Supporting Information), reaction times were not modeled in higher‐level analyses.

For each of the four primary higher‐level analyses, voxel‐wise nonparametric permutation testing (5,000 permutations) were implemented with FSL's PALM (Winkler, Ridgway, Webster, Smith, & Nichols, 2014). All statistical results are reported at *p* < .05 FWE cluster corrected (voxel‐wise height threshold *p* < .01). However, given the paucity of existing data regarding the effects of ECT and ketamine on emotion processing, results are also presented at *p* < .005 uncorrected (see Supporting Information). For visualization we used the Connectome Workbench platform (HCP, n.d.).

## 3 RESULTS

### 3.1 Demographic and clinical results

Age, sex, and education and clinical features of depression did not significantly differ between ECT and ketamine patients. Sex and age also did not differ between patients and HC, though education was greater in patients (Table 1). No significant differences for either of the clinical scales were observed between the ECT and ketamine samples at baseline. HDRS and SHAPS significantly decreased after both ketamine and ECT. However, DASS scores decreased significantly after ketamine only (Table 1).

### 3.2 Task‐fMRI results

#### 3.2.1 Functional mask

The average activation map for the all‐faces > objects contrast used to create the functional mask for subsequent analyses showed increased activation in the bilateral amygdala, right thalamus, right caudate, left cerebellum, and cerebellar vermis, and fusiform, insula, inferior frontal, DLPFC, precentral cortex, and precuneus (*p* < .05 FWE; Figure 2b).

#### 3.2.2 Main effects of treatment

**Figure 3:**
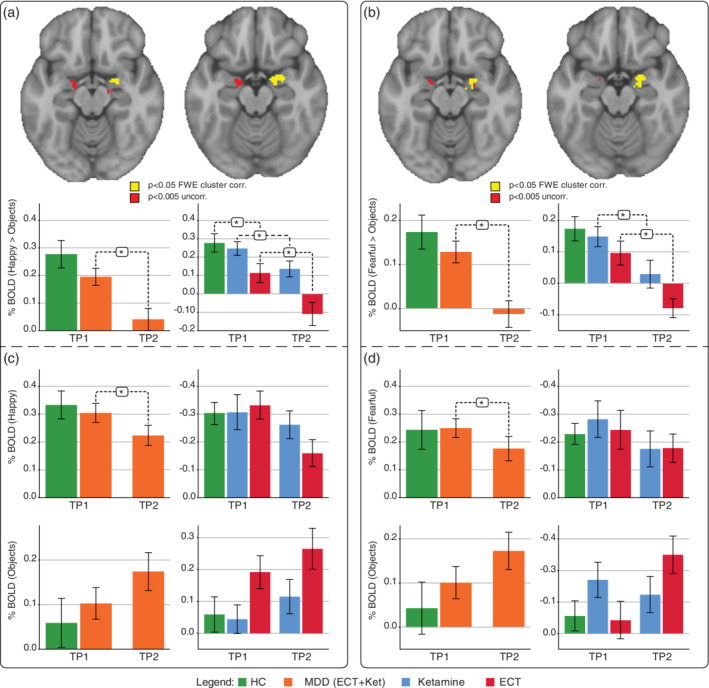
Effects of treatment across modality (ECT + Ketamine) for the happy > objects contrast (a and c) and fearful > objects contrast (b and d). (a) Significant clusters for the happy > objects contrast (top) and %BOLD signal derived from the corresponding amygdala ROI at TP1 (baseline) and TP2 (post‐treatment) averaged across (bottom right) and within treatment groups (bottom left); (b) Significant clusters for the for fearful > objects contrast (top) and %BOLD signal derived from the corresponding amygdala ROI at TP1 and TP2 averaged across (bottom right) and within treatment groups (bottom left). (c) %BOLD signal within the amygdala ROI (cluster derived from happy > objects contrast as in panel a) plotted for happy face stimuli (top) and objects (bottom) separately, again shown across (right) and within treatment groups (left); (d) %BOLD signal within the amygdala ROI (cluster derived from fearful > objects contrast as in panel a) plotted for fearful faces stimuli (top) and objects (bottom) separately, again shown across (right) and within treatment groups (left). %BOLD for neutral stimuli were not shown to change over time and are not plotted. ECT, electroconvulsive therapy; FWE, family wise error correction; HC, health controls; MDD, major depressive disorder patients

Comparing baseline (TP1) and post‐treatment (TP2) across all patients while controlling for treatment type revealed decreased activation the right amygdala (*p* < .05 FWE corrected) for both the fearful > objects and happy > objects contrasts after treatment. At more liberal threshold of *p* < .005 uncorrected, decreases were also observed for the left amygdala (Figure 3).

In the ketamine sample only, post‐treatment change in the fearful > objects contrast significantly correlated with %DASS and %SHAPS change in the right amygdala. Baseline amygdala activity did not predict measured clinical outcomes (Figure S1). The paired *t*‐test evaluating BOLD activity change before and after treatment for the neutral faces condition revealed no significant effects of time (*p* = .49).

#### 3.2.3 Differences in treatment‐related change across treatments

Within the larger emotional face processing network, changes in activation over time between ECT and ketamine treatment groups did not reach significance at *p* < .05 FWE. However, at *p* < .005 uncorrected, the fearful > objects contrast showed increased activity in the right DLPFC, and insula cortex after treatment in the ketamine sample in comparison to the ECT sample (see Figure S2).

#### 3.2.4 Correlations between treatment‐related change and clinical response

**Figure 4:**
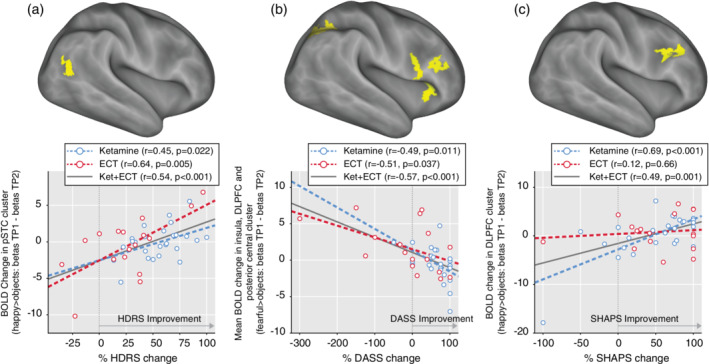
Associations between %BOLD signal change (TP1–TP2) and %change in clinical measures (*p* < .05 FWE cluster corrected). Significant clusters are shown in the top panel and corresponding linear regressions are shown below. (a) Superior parietal cluster BOLD change showed significant positive correlation with %HDRS change; (b) Insula, DLPFC, and posterior central BOLD change showed significant negative correlation with %DASS change; (c) DLPFC cluster BOLD change showed significant positive correlation with %SHAPS change. DASS, depression anxiety stress scale; DLPFC, dorsolateral prefrontal cortex; HDRS, Hamilton depression rating scale; SHAPS, Snaith–Hamilton pleasure scale

Examining associations with change in clinical outcome measures in the emotional face processing network, a cluster in the posterior superior temporal cortex (pSTC) showed a significant positive correlation between %HDRS change and BOLD change for the happy > objects contrast (TP1–TP2; Figure 4a). Three clusters in the right insula, right DLPFC and right postcentral cortex revealed a significant negative correlation between %DASS change after treatment, and BOLD change for the fearful > objects contrast (Figure 4b). Finally, a right DLPFC cluster showed a significant positive correlation between change in %SHAPS and BOLD response for the happy > objects contrast (TP1–TP2) (Figure 4c).

#### 3.2.5 Cross‐sectional group effects (HC VS MDD)

With *p* < .05 FWE correction, no differences were observed between HC and patients in the face processing network at baseline (see Figure S3 for uncorrected results).

## 4 DISCUSSION

This study investigated the neural effects of serial ketamine and ECT treatment for the processing of positive and negative emotionally valenced face stimuli in patients with TRD. To our knowledge, this study is the first to examine how two distinct and rapidly acting treatments for TRD (Duman et al., 2016; Murrough et al., 2013; Reed et al., 2018; Scheidegger, Henning, Walter, Lehmann, et al., 2016) similarly perturb emotional processing networks. Our results demonstrate that ketamine and ECT have similar effects on amygdalar response to affective faces, and reduce activation for both positively and negatively valenced stimuli despite their differences in initial neural targeting via pharmacotherapy or neurostimulation. However, results also suggest some distinctions in the broader emotional face processing network between fast‐acting treatment modalities.

At the molecular level, recent preclinical evidence suggests that ketamine's antidepressant effects extend beyond NMDA receptor blockade to include activation of α‐amino‐3‐hydroxy‐5‐methyl‐4‐isoxazolepropionic acid (AMPA) receptors and other signaling pathways (Aleksandrova, Phillips, & Wang, 2017; Zanos et al., 2016). Other novel findings show that ketamine's acute antidepressant effect may require opioid receptor activation (Amiaz, 2019; Williams et al., 2018). Though the molecular mechanisms associated with the antidepressant effects of ECT remain uncertain, preclinical studies point to neurotrophic (Duman et al., 2016) and neuroinflammatory processes (van Buel et al., 2015), and changes in neuro‐, synapto‐, dendro‐, and glio‐genesis, and dopaminergic and serotonergic signaling pathways (Baldinger et al., 2014; Chen, Madsen, Wegener, & Nyengaard, 2009; Madsen et al., 2000; Wennstrom, Hellsten, Ekdahl, & Tingstrom, 2003). Nonetheless, in aggregate, the antidepressant mechanisms for ECT, ketamine and other treatment modalities are expected to converge at the functional systems level to restore emotional processing deficits. At the same time, individual treatments may remain more suited for particular patients based on individualized clinical features. Research elucidating the relationships with downstream changes in neural circuitry and function, is thus important for understanding antidepressant response. In turn, this knowledge may allow better differentiation of systems‐level antidepressant mechanisms that may be both shared and distinct across treatment modalities.

### 4.1 Effects of treatment: Amygdala reactivity

Major depressive disorder is established to be associated with mood‐congruent negativity biases, where patients appear to process negative faces more rapidly and deeply than HC; processing of positive affective faces is also shown impaired (Stuhrmann et al., 2011). Amygdala reactivity to emotional stimuli has been consistently linked to mood‐congruent biases in depression during exposure to facial emotions, and to change with typical antidepressant treatment. In this investigation, we showed reduced amygdalar reactivity for both positive and negative stimuli after both ECT and ketamine treatment. These results are in line with decreases in amygdalar activity reported for the processing of negative stimuli in patients treated with standard antidepressants (Delaveau et al., 2011; Ma, 2015). Reductions in hippocampal‐amygdalar BOLD reactivity to negative emotional stimuli have also been reported in controls after single s‐ketamine administration (Scheidegger, Henning, Walter, Lehmann, et al., 2016) and patients with depression receiving ECT(Redlich et al., 2017), in accordance with our findings. The extant literature concerning the direction of change in amygdala responsivity to positive stimuli is less consistent (Stuhrmann et al., 2011) even among meta‐analytic studies (Delaveau et al., 2011; Ma, 2015). Here, results may be impacted by contrasts performed, medication type and single versus repeat antidepressant treatment (Ma, 2015). Notably, one prior study in MDD patients, reported an increase in dorsal striatum activation for positively valenced face stimuli after a single ketamine infusion (Murrough et al., 2015), which may differ from our results in patients receiving serial treatment.

Compatible with observations of similar amygdala effects in patients treated with ECT or solely with pharmacotherapy (Redlich et al., 2017), our findings suggest there is a nonspecific antidepressant treatment effect for both fast‐acting therapies at the level of the amygdala. Neural habituation is a mechanism shown in the amygdala after repeated emotional stimuli during consecutive runs in the same scanning session (Herry et al., 2007). However, habituation effects are no longer present over longer time intervals (~2 weeks or more) as demonstrated with test–retest measurements (Johnstone et al., 2005; Plichta et al., 2012; Plichta et al., 2014). In our study pre‐to‐post treatment time‐points are not continuous in time, but are separated by an interval of at least 2.5 weeks, ruling‐out the possibility of habituation effects. In follow‐up ROI analyses, we also demonstrate that BOLD activity in response to neutral faces does not change significantly over time, which further supports that results are not a reflection of habituation (Johnstone et al., 2005).

We did not observe differences in amygdalar activity when comparing patients to controls at baseline that have been reported in some prior cross‐sectional studies (Groenewold et al., 2013). However, at TP2 (after treatment) there is reduced BOLD activity in MDD patients comparatively to HC. These discrepant results in comparison to previous literature, where patients reveal hyper‐activity at baseline and normalization effects after treatment, could be due to methodological differences (comparing emotional faces or nonfacial stimuli against neutral faces or nonfacial stimuli), and/or due to the reduced statistical power for cross‐sectional patient‐control comparisons, which was not the main objective of this investigation. Rather, controls were included primarily to determine whether treatment effects indicate a normalization of brain activation. Notwithstanding, some previous literature suggests increased activity in the amygdala to sad, but not for fearful faces, which is in accordance with our results (Arnone et al., 2012).

### 4.2 System‐level mechanisms of ECT and ketamine and clinical outcomes

Using stringent correction thresholds, changes in BOLD response were not detected between treatments within the larger emotion face processing network. However, since no prior studies have investigated facial affect processing across rapidly acting antidepressants, results using a more liberal *p* < .005 threshold are provided in Supporting Information where statistical maps revealed a higher BOLD increase in the right DLPFC after ketamine in comparison to ECT (Figure S2). The DLPFC plays a role in emotion regulation and cognitive control, and previous investigations have reported decreased average global brain connectivity in the DLPFC and decreased connectivity of the DLPFC to limbic regions after ECT, which may play a role in restored emotional processing as well as cognitive side effects (Perrin et al., 2012). Studies assessing the role of the PFC in emotional processing in association with the antidepressant effects of ketamine are sparse. However, the examination of emotionally valenced attention (Reed et al., 2018) and implicit and explicit emotion processing (Reed et al., 2019), revealed changes in neural response in cortical association regions including the prefrontal cortex pre‐to‐post treatment in patients. Still, further studies are required to better understand if differences in DLPFC activity diverge across treatments. Treatment design differences between ketamine and ECT patient populations may also affect BOLD measures. However, the main aim of this study was to investigate mechanisms at the system level for within and between treatments after a comparable treatment series (index series for ECT and serial ketamine infusions) understanding that the time scale of response differs between treatment modalities.

Emotion processing is linked with clinical features of depression. Notably, in this study, FWE‐corrected maps revealed an association between change in BOLD response to positive facial stimuli in the pSTC and change in mood ratings (Figure 4). The pSTC, a multimodal association region involved in body and facial emotion processing (Jastorff, Huang, Giese, & Vandenbulcke, 2015; Peelen, Atkinson, & Vuilleumier, 2010) and known to form part of a network with connections to the amygdala, insula and PFC, and visual and language areas (Goldin, McRae, Ramel, & Gross, 2008; Sarkheil, Goebel, Schneider, & Mathiak, 2013), has previously been linked to abnormally high BOLD activity to emotional faces in MDD (Pulcu, Zahn, & Elliott, 2013). Change in this network could thus underlie both symptom improvement and restored emotion processing function. Additionally, results revealed significant correlations for both ECT and ketamine groups between BOLD changes with treatment to negative emotional stimuli in clusters in the right DLPFC, insula, and posterior central cortex with change in anxiety scores (BOLD increases correlated with DASS value decreases). Frontal regions are consistently found to show decreased activation in MDD (Smoski et al., 2015; Stuhrmann et al., 2011). Anxiety has been connected to deficits in activation in ventral cingulate and amygdala as well as with compensatory effects in the DLPFC (Oathes, Patenaude, Schatzberg, & Etkin, 2015). Negativity bias in depression may lead to insufficient top‐down inhibitory control from frontal cortical regions to limbic and striatal regions (Domschke et al., 2015). By normalizing frontal BOLD activity, top–down regulation of limbic regions may reset negativity biases.

Anhedonia, defined as the inability to feel pleasure, is one of the most prevalent MDD symptoms. However, standard antidepressant treatments are shown as less effective for reducing anhedonia (Hoflich, Michenthaler, Kasper, & Lanzenberger, 2019; Lally et al., 2014). Recently, ketamine has been shown to alleviate anhedonic symptoms (Hoflich et al., 2019). Accordingly, our results show that the change in anhedonia scores significantly correlate with BOLD changes to positive emotional stimuli in a DLPFC cluster (BOLD decreases correlate with SHAPS values decreases). When ketamine and ECT are analyzed separately in this cluster, results suggest that the correlation is mostly driven by ketamine and not by ECT (Figure 4). Previous studies have linked anhedonia to defective positive affect processing and reward systems, which include ventromedial prefrontal cortex, amygdala, and ventral striatum, in depression (Keedwell et al., 2005). Previous studies have shown that increased CADSS score at 40 min are predictive of treatment outcomes (Luckenbaugh et al., 2014). Investigating possible associations between acute CADSS scores and BOLD measures would thus provide further information about mechanistic effects of ketamine. However, we only acquired these scales after 1 hr of each infusion at which point most all subjects did not show side effects.

### 4.3 Limitations

Since the primary objective of this study was to investigate longitudinal treatment effects, a limitation is that we may have been less able to detect patient‐control differences in less powerful cross‐sectional comparisons, which did not survive multiple comparisons correction. Nonetheless, at lower thresholds, patients showed hyper‐responsivity to affective faces in the cerebellum compared to HC (see Supporting information). Additional study limitations include that HCs were not measured twice. However, previous studies have shown that amygdalar habituation to fearful faces do not persist over independent session separated by time, ruling‐out possible habituation effects in our sample assess >2 weeks apart. Also, ECT patients were tapered‐off of antidepressant medications prior to treatment, whereas the ketamine participants were allowed to continue on stable antidepressant medication, which may have impacted findings. It is important to note that the focus of this investigation was on change in neural response over time where subjects serve as their own controls. Finally, treatment was administered using a naturalistic design. Consequently, we cannot fully exclude placebo effects or that patient groups differed at baseline, though notably, this study focused on neurofunctional changes and relationships with clinical outcomes, rather than group differences in response measures themselves arguing against the possibility of placebo effects that presumably do not perturb functional circuitry to the same extent as active treatment. Importantly, the same inclusion/exclusion criteria were used for TRD patients in both treatment groups (with the exception of any subject‐specific contraindications to ketamine) and groups did not differ with respect to any measured clinical features (e.g., disease severity, treatment history, duration of illness).

## 5 CONCLUSION

Both ketamine and ECT interventions result in decreases in amygdalar reactivity during the processing of positive and negative stimuli, which suggests downstream antidepressant mechanisms overlap at the higher functional systems level. Less pronounced differences were observed between fast‐acting treatments in the DLPFC and insula. Notably, task‐related changes in BOLD response in the inferior parietal cortex were significantly associated with overall change in mood, and BOLD change in the prefrontal cortex was significantly associated with symptoms of anxiety and anhedonia suggesting neural changes may serve as biomarkers for particular depressive symptoms.

## CONFLICT OF INTEREST

The authors have no conflict of interest to declare.

## ETHICS STATEMENT

The authors assert that all procedures contributing to this work comply with the ethical standards of the relevant national and institutional committees on human experimentation and with the Helsinki Declaration of 1975, as revised in 2008.

## Supporting information


**Appendix S1:** Supporting information

## Data Availability

Data sharing is not applicable to this article as no new data were created or analyzed in this study.
