# Supplementary material for: Modulation of amygdala reactivity following rapidly acting interventions for major depression
Source: Hum Brain Mapp. 2020 Mar 1;41(7):1699–710. doi: 10.1002/hbm.24895 (PMC7268016; doi:10.1002/hbm.24895)
Supplement: Supplementary file 1 — Appendix S1: Supporting information [file HBM-41-1699-s001.docx]

# Supplementary Material:

# Supplementary Methods

## Participants

Patients eligible for ketamine treatment (n=27) were recruited from clinician referral, clinicaltrials.gov (NCT02165449) or targeted advertisements. Ketamine patients were also screened to ensure no prior psychotic reactions to medications, alcohol or illicit substances in the past, and for any other physical or clinical contraindications to ketamine. ECT patients (n=17) were recruited from individuals already scheduled to receive ECT therapy at the Resnick Neuropsychiatric Hospital at the University of California, Los Angeles (UCLA). Non-depressed control subjects were recruited from the same geographical area in Los Angeles via online and paper advertisements.

Exclusion criteria for all patient and control participants included any serious or unstable medical or neurological condition, current substance abuse or dependence (ascertained by laboratory testing) or substance abuse history within the preceding 3-months, current or past history of psychosis, schizoaffective disorder or schizophrenia, mental retardation or other developmental disorder, diagnosis of dementia of any type, and any contraindication to scanning (e.g., metal implants or claustrophobia). Supplementary Table 1 summarizes the number of patients in the ketamine group taking different types of medication. ECT patients were tapered off of medication.

**Supplementary Table 1-** Number of patients in the ketamine group taking medication (divided into different categories).

## Image Acquisition and preprocessing

Structural scans included a T1-weighed (T1w) multi-echo MPRAGE (voxel size (VS)=0.8mm isotropic; repetition time (TR)=2500ms; echo time (TE)=1.81:1.79:7.18ms; inversion time (TI)=1000ms; flip angle (FA)=8.0^o^; acquisition time (TA)=8:22min), and a T2-weighted (T2w) acquisition (VS=0.8mm isotropic; TR=3200ms; TE=564ms; TA=6:35min), both with real-time motion correction.

After preprocessing, the functional images were further denoised using FSL’s FIX (<https://fsl.fmrib.ox.ac.uk/fsl/fslwiki/FIX>), where independent components analysis (ICA) and a custom built training dataset (n=25) was used to identify and remove components representing noise in the data.

## Emotional Faces Functional Imaging Task

For each of the 2 functional runs, 3 blocks of each condition were presented, and each block consisted of 6 trials (3 s each). Interleaved between 3 consecutive blocks of the 3 different conditions, a resting period using a fixation cross of 16 s was presented (for a total of 4 resting periods). Each run lasted 4 min and 41 s with 338 volumes. The stimulus sequence was randomized across subjects.

**Task fMRI Analysis**

### Regions-of-Interest Analysis

Post-hoc analyses were performed to visualize patterns of regional signal change for clusters that showed significant activation (p<0.05 FWE) when comparing baseline (TP1) to post-treatment (TP2) for the happy > objects and fearful > objects contrasts. For each analysis a functional mask was generated by binarizing the BOLD activation map at the defined threshold. Beta values were then extracted from the generated regions-of-interest (ROIs) for both contrasts, and for the individual stimulus conditions (happy, fearful and objects). The extracted beta values were averaged for each ROI and divided by the mean fMRI value in order to obtain percent signal change values per ROI. GLMMs evaluated effects of time within patient groups, and two-sample t-tests compared treatment groups and all patients compared to controls at baseline.

Follow-up ROI analysis for the significant amygdalar clusters obtained for fearful>objects and happy>objects contrasts were used to evaluate correlations between change in clinical measures and change in BOLD response with treatment (TP1-TP2), and differences between diagnostic group at baseline. To evaluate possible habituation effects for amygdala reactivity, differences in BOLD activity in amygdalar clusters showing treatment effects were evaluated pre- and post-treatment for the neutral faces exclusively.

# Supplementary Results

## Task fMRI Results

### Task Performance

FMRI task performance was evaluated for use as a potential covariate for longitudinal and cross-sectional comparisons. No significant differences in reaction times were detected between MDD patients (Mean (M)=0.84, SD=0.32) and healthy controls (HC) (M=0.744; SD=0.39) at baseline, t(73)=1.22, p=0.26. Similarly, there were no significant differences in reaction times for the ECT (M=0.92, SD=0.29) and the ketamine samples (M=0.80, SD=0.34) at baseline, t(41)=-1.22, p=0.23. In addition, no significance effects of time, F(41)=0.57, p=0.45), or treatment group by time interactions (F(41)=0.03, p=0.86) was observed. Since significant main effects or interactions were absent, reaction times were not modeled in higher-level analyses.

### Clinical Correlates with Treatment-related changes in Amygdalar Responsivity:

When ketamine and ECT were analyzed separately, change in BOLD (TP1-TP2) after ketamine treatment in the right amygdala cluster for the fearful>objects contrast significantly correlated with change in %DASS and %SHAPS scores [Supplementary Figure 1].

**Supplementary Figure 1**. Correlations between change in BOLD response (TP1 – TP2) for amygdalar clusters showing overall treatment effects and clinical improvement (% change) in depressive symptoms (HDRS scale) (B, F); anxiety (DASS scale) (C,G); and anhedonia (SHAPS scale) (D,H). (A-D): Right amygdala cluster BOLD change correlation results for happy>objects contrast; (E-F): Right amygdala cluster BOLD change correlation results for fearful>objects contrast. For all plots, the gray line represents the mean regression line for all MDD patients (ECT+Ket), the blue dashed lines represent the regression lines for ketamine patients and the red dashed lines represent the regression lines for ECT patients.

### Differences Between ECT and Ketamine Treatments:

Uncorrected maps at p<0.005 showed higher BOLD response increases in the DLPFC, insula, and caudal middle frontal cortex after ketamine treatment in comparison to ECT treatment. Follow-up ROI analysis revealed that in these regions BOLD response is increasing after ketamine treatment and significantly decreasing after ECT treatment [Supplementary Figure 2].

**Supplementary Figure 2.** Independent sample t-test between BOLD changes after ECT and BOLD changes after ketamine treatment (p<0.005 uncorrected). A) Clusters (DLPFC, insula and caudal middle PFC) that showed greater increases in BOLD response for ketamine in comparison to ECT treatment for the fearful>objects contrast. B) BOLD values extracted from DLPFC cluster; C) BOLD values extracted from insula cluster; D) BOLD values extracted from caudal middle PFC cluster.

### Cross-Sectional Group Effects Between HC and MDD Patients:

A 2-sample t-test revealed significantly higher BOLD response for happy>objects contrast in the cerebellum vermis and in the left lobule VII of the cerebellum in MDD patients at baseline (TP1) comparatively to HC. ROI analysis of these significant cerebellar clusters showed that BOLD response significantly decreases after treatment for the ketamine sample for happy>objects contrast and for the happy condition versus rest. No significant changes were observed for the objects condition versus rest, and the ECT sample did not show significant changes with treatment for any of the contrasts and individual conditions [Supplementary Figure 3].

**Supplementary Figure 3.** Cross-Sectional Effects (independent sample t-test) between HC and MDD patients (ECT + ketamine groups) at baseline (TP1) (p<0.01 uncorrected). A) Cerebellum cluster that showed hyper-activation at TP1 for MDD patients in comparison to HC for happy>objects contrast; B) BOLD values extracted from cerebellum cluster for happy>objects contrast; C) BOLD values extracted from the cerebellum cluster for the happy condition; D) BOLD values extracted from the cerebellum cluster for the objects condition.
